# Patterns of mental health service use among perpetrators of domestic homicide: descriptive study of Domestic Homicide Reviews in England and Wales

**DOI:** 10.1192/bjb.2023.91

**Published:** 2024-12

**Authors:** Poppy MacInnes, Marilia A. Calcia, Michela Martinuzzi, Cora Griffin, Siân Oram, Louise M. Howard

**Affiliations:** 1 King's College London, London, UK; 2 South London and Maudsley NHS Foundation Trust, London, UK

**Keywords:** Domestic violence and abuse, domestic homicide, domestic homicide reviews, family homicide, intimate partner homicide

## Abstract

**Aims and method:**

We used data from Domestic Homicide Reviews (DHRs) to describe the patterns of mental health service use by perpetrators of domestic homicide in England and Wales. In 186 DHR reports we compared the characteristics of perpetrators who accessed mental health services with those of perpetrators who did not. Separate analyses were conducted for perpetrators of intimate partner homicide (IPH) and family homicide.

**Results:**

Over two-thirds (64.5%, *n* = 120) of the perpetrators had accessed mental healthcare before the homicide. Perpetrators of IPH who had used mental health services compared with those who had not were more likely to have a history of substance misuse, contact with the criminal justice system and a history of self-harm or suicide attempts.

**Clinical implications:**

Our findings support the need for health services, particularly mental health, addictions and primary care, to take an assertive role in the prevention of domestic violence and abuse by identifying patients who are potential perpetrators of domestic violence and abuse.

Domestic violence and abuse (DVA) victimisation is a major public health issue: it affected 2.4 million adults aged 16 and above (1.7 million women and 699 000 men) in England and Wales in the year ending March 2022.^1^ Worldwide, it has a lifetime prevalence of over one-quarter among women aged 15–49 years who have ever been in a relationship.^2^ Domestic violent crime rates against women in the UK have been rising since 2008, whereas violent crime against men has been falling.^3,4^ The physical and mental health effects of DVA on survivors have been well documented.^5–8^ In the UK, DVA includes intimate partner violence and adult family violence (AFV) and it is defined as ‘any incident or pattern of incidents of controlling, coercive or threatening behaviour, violence or abuse between those aged 16 or over, who are or have been intimate partners or family members regardless of gender or sexuality’.^9^

In 2011, the UK introduced statutory Domestic Homicide Review (DHR) reports, which are multi-agency reviews into deaths caused by DVA or neglect in individuals aged 16 or over by a family member or a current or ex-partner. They are carried out by Community Safety Partnerships (CSPs) made up of representatives of local services with the aim of learning from each homicide and preventing future incidents. Similar multi-agency reviews are undertaken in the USA, Canada, Australia, New Zealand and Portugal.^10^

Violent offending has been linked to common mental disorders and severe mental illness.^11–15^ Systematic reviews have demonstrated that mental disorders are associated with increased risk of physical violence against partners,^6,16,17^ and previous analyses of DHRs have shown a high prevalence of mental health problems among perpetrators of domestic homicide. In a sample of 141 DHR reports, 64% of perpetrators had mental health problems, as did 78% of perpetrators in a sample of 66 adult family homicide reports.^10,18^ Studies from the UK Home Office and third-sector organisations have also highlighted the high prevalence of mental health service use among perpetrators of domestic homicides.^19–21^

Mental health service use encompasses a wide range of diagnoses and interventions. Research to date has not provided sufficient clarity on the type of mental health needs or the patterns of service use among perpetrators of domestic homicide, or on how perpetrators who used mental health services differ from those who did not. Planning and delivering interventions therefore remains challenging.

This study aims to describe the patterns of lifetime mental health service use among perpetrators of domestic homicide before the offence, based on data from DHR reports published between April 2011 and December 2020. We compare the characteristics of perpetrators who accessed mental health services pre-homicide with those of perpetrators who did not.

## Method

This is a descriptive study of publicly available Domestic Homicide Reviews (DHRs) that have undergone quality assurance by the UK Home Office and were published on local authority or Community Safety Partnership websites.

### Sampling

Data collection was conducted in June 2020 via searches of the websites for 314 Community Safety Partnership or Local Authorities (LA) in England and Wales from the introduction of DHRs in 2011 until the date of the search. We did not include cases in which only the executive summary was available, as those did not usually include the details on mental health service use required for this study. We obtained 186 DHR overview reports.

### Inclusion criteria

All overview reports where a domestic homicide (adult family homicides and intimate partner homicides) had been perpetrated by an adult (defined by the Home Office DHR criteria as a person aged 16 or over) available online at the time of data collection (June 2020).

### Exclusion criteria

Reports in which the victim had taken their own life as a result of DVA or in which the perpetrator was aged under 16 at the time of the offence.

Reports in which the relationship between victim and perpetrator was not of intimate partner or family (e.g. employee/employer living in the same household or another cohabiting person who was not in an intimate relationship or family).

Reports for which only the executive summary could be found.

### Data extraction

All reports were read fully by at least one member of the research team (P.M., M.A.C., C.G. or M.M.) who were tasked with data extraction. M.A.C. and P.M. developed an extraction form in Microsoft Excel with demographic and clinical characteristics of interest. The form was piloted on 20 reports. Data extraction was checked for accuracy by M.A.C. on 10 of the reports read by P.M., C.G. and M.M.

Data on perpetrators’ mental health service use prior to the homicide were collected. Mental health service use was defined as instances in which the perpetrator had their mental health assessed by a healthcare professional in primary care (including general practice and other services), secondary mental healthcare, private mental healthcare or addictions services. Referrals to mental health services that the perpetrator never attended were not included.

### Data analysis

Descriptive statistics were calculated to report on: perpetrators’ demographic characteristics; perpetrators’ history of psychiatric diagnosis, primary and secondary mental health service use, and substance misuse (including substance misuse diagnoses or any mention of problematic use of alcohol or non-prescribed psychoactive substances before or at the time of the homicide); forensic history; whether the perpetrator or victim had children under the age of 16; history of DVA perpetration by the perpetrator in any relationship prior to the homicide; history of suicidality and self-harm by the perpetrator, and cases of attempted or completed suicide after the homicide. We also extracted data on victims’ gender.

We compared data of perpetrators who had a lifetime history of any mental health service use within the review period with data of those who had no such history and conducted separate analyses for the intimate partner homicide (IPH) and family homicide groups. The statistical tests used were chi-squared, Fisher's exact and unpaired *t*-tests on Stata17 software for MacOS.

### Ethics approval

Ethics approval for this study was not required as all information used is readily available online.

## Results

Characteristics of the sample are reported in Table 1. Of 186 DHR reports, 150 were for IPH (80.7%) and 36 were for family homicide (19.4%). Eleven reports detailed cases with more than one victim: ten were IPH in which adult family members (*n* = 2), an adult family member and a child (*n* = 1), children (*n* = 6) or the victim's new partner (*n* = 1) were killed in the same incident as the intimate partner. There was one family homicide with two victims (mother and adult sister). Owing to their small number (*n* = 3), homicides in which both intimate partners and adult family members were killed were grouped with IPH for analysis.

**Table 1:** Characteristics of the whole sample of Domestic Homicide Review (DHR) reports and a comparison between perpetrators of intimate partner homicide and perpetrators of adult family homicide

	All DHR reports	Intimate partner homicide reports (*k* = 150)	Adult family homicide reports (*k* = 36)	*P* (statistical analysis used)^a^
Victim's gender	*n* = 200 victims	*n* = 162 victims	*n* = 38 victims	***P* < 0.001** (Fisher's exact)
Adult male	41 (20.5%)	23 (14.2%)	18 (47.3%)	
Adult female	151 (75.5%)	131 (80.8%)	20 (52.6%)	
Child ≤16 years	5 girls (2.5%); 2 boys (1%), 1 gender not stated (0.5%)	8 (4.9%): 5 girls (3.1%); 2 boys (1.2%); 1 (0.6%) gender not specified	0	
Homicide–suicide (*n* = 186)	40 (22%)	36 (24%)	4 (11.1%)	*P* = 0.115 (Fisher's exact)
Completed suicide	10 (5.4%)			
Attempted suicide	30 (16.1%)			
Perpetrator's gender (*n* = 186)				*P* = 0.770 (χ^2^)
Male	165 (88.7%)	132 (88%)	33 (91.7%)	
Female	21 (11.3%)	18 (12%)	3 (8.3%)	
Perpetrator's age (*n* = 130), years: mean (range)	42 (19–86)	43.9 (19–86)^b^	35.6 (19–73)^c^	***P* = 0.007** (unpaired *t*-test)
Perpetrator's ethnicity (*n* = 133)		*n* = 109 perpetrators	*n* = 24 perpetrators	*P* = 0.974 (Fisher's exact)
White	88 (66.7%)	72 (66%)	17 (70.1%)	
Asian/Asian British	21 (15.9%)	18 (16.5%)	3 (12.5)	
Black/Black British	17 (12.9%)	14 (12.8%)	3 (12.5%)	
Other	4 (3%)	5 (4.6%)	1 (4%)	
History of self-harm or suicidality before the homicide (*n* = 186)	81 (43.5%)	67 (44.7%)	16 (44.4%)	*P* = 0.981 (χ^2^)
History of any mental health service use (*n* = 186)	120 (64.5%)	90/150 reports (60%)	30/36 reports (83.3%)	***P* = 0.009** (χ^2^)
Type of service used				
Secondary care (mental health or addictions services)	109 (85%)	81 (54%)	28 (77.8%)	
General practice only	11 (6%)	9 (6%)	2 (5.5%)	
Type of mental health service used^d^	*n* = 120 perpetrators	*n* = 90 perpetrators	*n* = 30 perpetrators	n.a.
A&E/mental health liaison and/or place of safety	33 (27%)	28 (31.1%)	5 (16.7%)	
Community mental health team	47 (39%)	27 (30%)	20 (66.7%)	
Addictions services	30 (25%)	25 (27.8%)	5 (16.7%)	
Talking therapies	32 (27%)	22 (24.4%)	10 (33.3%)	
In-patient mental health ward	27 (22%)	19 (21.1%)	8 (26.7%)	
Crisis/home treatment team	8 (6%)	6 (6.7%)	2 (6.7%)	
Child and adolescent mental health services	8 (6%)	4 (4.4%)	4 (13.3%)	
Forensic or custody services	10 (8%)	5 (5.5%)	5 (16.7%)	
Other	5 (4%)	5 (5.5%)	0	
Perpetrator's diagnoses (mental health service use group only)^d^	120 perpetrators	90 perpetrators	30 perpetrators	n.a.
Mood/anxiety				
Depression or adjustment disorder	53 (44.2%)	40 (44.4%)	13 (33.3%)	
Anxiety disorders	30 (25%)	22 (24.4%)	8 (26.7%)	
‘Low mood’	4 (3.3%)	3 (3.3%)	1 (3.3%)	
Bipolar affective disorder	3 (2.5%)	2 (2.2%)	1 (3.3%)	
Depression with psychotic symptoms	3 (2.5%)	3 (3.3%)	0	
Personality disorders				
EUPD	7 (5.8%)	5 (5.5%)	2 (6.7%)	
Dissocial personality disorder	3 (2.5%)	2 (2.2%)	1 (3.3%)	
Personality difficulties/‘possible personality disorder’	2 (1.7%)	1 (1.1%)	1 (3.3%)	
Substance use disorders (excluding drug-induced psychosis):	7 (5.8%)	6 (6.7%)	1 (3.3%)	
Psychotic disorders				
Schizophrenia	10 (8.3%)	3 (3.3%)	7 (23.3%)	
Drug-induced psychosis	2 (1.7%)	1 (1.1%)	1 (3.3%)	
Psychosis not otherwise specified	4 (3.3%)	2 (2.2%)	2 (6.7%)	
Transient acute psychosis	1 (0.8%)	1 (1.1%)	0	
Delusional disorder	1 (0.8%)	1 (1.1%)	0	
Other mental disorders:				
Dementia	3 (2.5%)	3 (3.3%)	0	
Intellectual disability	1 (0.8%)	1 (1.1%)	0	
Eating disorder	1 (0.8%)	1 (1.1%)	0	
ADHD	2 (1.7%)	1 (1.1%)	1 (3.3%)	
Conduct disorder	2 (1.7%)	1 (1.1%)	1 (3.3%)	
Autism	1 (0.8%)	0	1 (3.3%)	
Mental disorder caused by head injury	1 (0.8%)	1 (1.1%)	0	
No diagnosis	25 (20.8%)	21 (23.3%)	4 (13.3%)	
Perpetrator's last contact with mental health services (mental health service use group only)	*n* = 105	*n* = 77^e^	*n* = 28^f^	*P* = 0.257 (Fisher's exact)
<1 month	36 (34%)	28 (36.3%)	8 (28.5%)	
1–11 months	36 (34%)	28 (36.3%)	8 (28.5%)	
1–5 years	30 (28%)	18 (23.3%)	12 (42.8%)	
>5 years	3 (3%)	3 (3.9%)	0	
History of substance misuse (*n* = 186)	118 (63.4%)	92 (61.3%)	26 (72.2%)	*P* = 0.223 (χ^2^)
History of DVA perpetration (*n* = 186)	130 (69.9%)	110 (73.3%)	20 (55.5%)	***P* = 0.037** (χ^2^)
Perpetrator/victim cohabited at time of the homicide (*n* = 186)	122 (65.6%)	99 (66%)	23 (63.9%)	*P* = 0.8463 (Fisher's exact)
History of contact with police or criminal justice system (*n* = 186)	133 (71.5%)	107 (71.3%)	26 (72.2%)	*P* = 0.915 (Fisher's exact)
Perpetrator/victim had children aged 16 or under (incl. unborn children) (*n* = 186)	82 (44.1%)	73 (48.7%)	9 (25%)	***P* = 0.010** (χ^2^**)**

A&E, accident and emergency; EUPD, emotionally unstable personality disorder; ADHD, attention-deficit hyperactivity disorder; DVA, domestic violence and abuse; n.a., not applicable; incl., including.

a. *P-*values indicate statistically significant differences between the intimate partner homicide and family homicide groups. Bold denotes significance at *P* < 0.05.

b. Reported in 112/150 reports (74.7%).

c. Reported in 28/36 reports (77.8%).

d. For the ‘Type of mental health service’ and ‘Perpetrator's diagnoses’ cells, the categories listed are not mutually exclusive, as many individuals used more than one type of service and/or received more than one diagnosis. Statistical testing was not conducted for these.

e. Information not available in 13 reports.

f. Information not available in 2 reports.

The majority of perpetrators (*n* = 165, 88.7%) were male and most victims (78%) were female (75.5% adult females and 2.5% girls). Demographic details on age and ethnicity were not provided in all reports. In the 132 reports where ethnicity was provided, 88 perpetrators (66.7%) were White British. Two-thirds (65.6%) lived with the victim at the time of the homicide, and in two-fifths of cases (44.1%) either the victim or the perpetrator had children under the age of 16.

A history of self-harm or suicidal thoughts or behaviour before the homicide was reported for 43.5% of perpetrators, and 21.5% of the homicides were followed by suicide attempts or completed suicide of the perpetrator. Over two-thirds of perpetrators (64.5%) had accessed mental healthcare and a similar number (63.4%) had a history of substance misuse; alcohol was the most commonly misused substance (52.2%), followed by cannabis (16.2%).

The majority of perpetrators (70.4%) had a history of DVA perpetration before the homicide. However, the reports indicated that these histories were not always known to agencies such as police or healthcare services. Almost two-thirds (65%) had a history of contact with the police or the criminal justice system, although not always for DVA-related incidents.

In Table 1 we compared data on perpetrators of IPH with perpetrators of family homicide. There were 162 victims in 150 IPH reports (including 8 children and 4 adults other than the partner/victim) and 38 victims in 36 family homicide reports. There were no child homicides in the family homicide group. The relationships of perpetrator to victim in family homicide were son (*n* = 23), grandson (*n* = 2), brother *n* = 8), daughter (*n* = 2), sister (*n* = 1) and other (*n* = 2: 1 boyfriend of victim's daughter and 1 son of victim's partner).

IPH perpetrators were, on average, older than family homicide perpetrators (mean age 43.9 years and 35.6 years respectively; *P* = 0.007). Perpetrators in both groups were primarily male (88% in the IPH group, 91.7% in the family homicide group) and most victims were female (80.8% in the IPH group, 52.6% in the family homicide group; *P* < 0.0001). The IPH group was more likely to have children under the age of 16 than the family homicide group (48.7% *v.* 25%; *P* = 0.014).

In many cases, individuals used more than one mental health service and/or were given more than one diagnosis during the period reviewed by the DHR reports; all diagnoses given in the reports and all services accessed by perpetrators were included in our analysis. Common mental disorders such as depression and anxiety were the most frequent diagnoses in both groups. A larger proportion of perpetrators in the family homicide mental health service use (FH-MH) group had a diagnosis of schizophrenia (*n* = 7, 23.3%) than in the IPH mental health service use (IPH-MH) group (*n* = 3, 3.3%).

Type of service used and period of service use in relation to the homicide are reported in Table 1. The IPH-MH group had a higher rate of use of accident and emergency and general hospital mental health liaison services (31.1%, compared with 16.7% in the FH-MH group), while the use of community mental health teams in the FH-MH group was more than twice that of the IPH-MH group (66.7% *v.* 30%).

We compared the characteristics of perpetrators who accessed mental health services within the review period and those who did not in Table 2 (for IPH) and Table 3 (for family homicide).

**Table 2:** Mental health (MH) service use versus no mental health service use (nMH) among perpetrators of intimate partner homicide (IPH)

	History of mental health service use (IPH-MH) (*n* = 90 perpetrators)	No history of mental health service use (IPH-nMH) (*n* = 60 perpetrators)	*P* (statistical test used)^a^
Victim's gender	*n* = 97	*n* = 65	***P* = 0.045** (Fisher's exact)
Adult female	74 (76.3%)	57 (87.7%)	
Adult male	19 (19.6%)	4 (6.1%)	
Child (male or female)	4 (4.1%)	4 (6.1%)	
Homicide–suicide			*P* = 0.876 (χ^2^)
Completed suicide	17 (18.9%)	10 (16.7%)	
Attempted suicide	5 (5.5%)	4 (6.7%)	
Perpetrator's gender			***P* = 0.040** (Fisher's exact)
Male	75 (83.3%)	57 (95%)	
Female	15 (16.7%)	3 (5%)	
Perpetrator's age (where stated), years: mean (range)	42.17 (19–82)	46.16 (21–86)	*P* = 0.1531 (unpaired *t*-test)
Perpetrator's ethnicity	*n* = 61	*n* = 48	*P* = 0.223 (Fisher's exact)
White	45 (73.7%)	27 (56.2%)	
Asian	9 (14.7%)	9 (18.7%)	
Black	5 (8.2%)	9 (18.7%)	
Other	2 (3.3%)	3 (6.2%)	
History of self-harm or suicidality before homicide	56 (62.2%)	9 (15%)	***P* < 0.001** (χ^2^)
History of substance misuse	62 (68.9%)	30 (50%)	***P* = 0.020** (χ^2^)
History of DVA perpetration	69 (76.7%)	39 (65%)	0.119 (χ^2^)
Perpetrator and victim cohabited at the time of the homicide	61 (67.8%)	38 (63.3%)	*P* = 0.573 (χ^2^)
History of contact with police or criminal justice system	71 (78.9%)	36 (60%)	***P* = 0.012** (χ^2^)
Perpetrator or victim had children under 16	42 (46.7%)	31 (51.7%)	*P* = 0.548 (χ^2^)

DVA, domestic violence and abuse.

a. *P-*values indicate statistically significant differences between the two groups. Bold denotes significance at *P* < 0.05.

**Table 3:** Mental health (MH) service use versus no mental health service use (nMH) among perpetrators of family homicide (FH)

	History of service use history (FH-MH) (*n* = 30)	No history of mental health service use (FH-nMH) (*n* = 6)
Victim's gender	*n* = 32	*n* = 6
Male	14 (43.7%)	4 (66.7%)
Female	18 (56.2%)	2 (33.3%)
Suicide–homicide		
Completed suicide	3 (10%)	0
Attempted suicide	1 (3.3%)	0
Perpetrator's gender		
Male	27 (90%)	6 (100%)
Female	3 (10%)	0
Perpetrator's age, years: mean (range)	36.9 (19–73)	29.6 (19–51)
Perpetrator's ethnicity	*n* = 20	*n* = 4
White British	15 (75%)	2 (50%)
Asian	3 (15%)	0
Black	1 (5%)	2 (50%)
Other	1 (5%)	0
Perpetrator's relationship to victim	*n* = 31	*n* = 6
Son	21 (67.7%)	1 (16.7%)
Brother/half-brother	4 (12.9%)	4 (66.7%)
Grandson	2 (6.4%)	0
Daughter	2 (6.4%)	0
Sister	1 (3.2%)	0
Other	1 (3.2%)	1 (16.7%)
Perpetrator had history of self-harm or suicidality before the homicide	16 (53.3%)	0
Perpetrator had substance use history	22 (84.6%)	4 (66.7%)
Perpetrator had history of DVA perpetration	16 (53.3%)	4 (66.7%)
Perpetrator and victim cohabited at the time of the homicide	18 (60%)	5 (83.3%)
Perpetrator had history of contact with police or criminal justice system	21 (70%)	5 (83.3%)
Perpetrator or victim had children aged 16 or under	8 (26.7%)	1 (16.7%)

DVA, domestic violence and abuse.

In the IPH group (Table 2), although most perpetrators were male, there was a higher proportion of male perpetrators in the no mental health service use (IPH-nMH) group (*n* = 57, 95%) than in the mental health service use (IPH-MH) group (*n* = 75, 83.3%; *P* = 0.040). The difference in the gender distribution of victims was also statistically significant; although most victims in both groups were female, the IPH-MH group had a higher proportion of male victims (*n* = 19, 19.6%) than the IPH-nMH group (*n* = 4, 6.1%). There were 4 child homicides in each IPH group (4.1% victims in the IPH-MH group and 6.1% in the IPH-nMH group).

IPH-MH perpetrators were more likely to have a history of substance misuse (68.9% *v.* 50%; *P* = 0.020), contact with the police or criminal justice system (78.9% *v.* 60%; *P* = 0.012) and a history of self-harm or suicidality than the IPH-nMH group (62.2% *v.* 15%; *P* < 0.001); rates of actual or attempted suicide after homicide did not differ significantly between the two groups. More individuals in the IPH-MH group had a history of DVA perpetration than in the IPH-nMH group (76.7% *v.* 65%), but the difference was not statistically significant (*P* = 0.119).

Of the 36 perpetrators of family homicide, most (*n* = 30, 83.3%) had a history of mental health service use (FH-MH). No statistical testing was conducted to compare the two groups of family homicide perpetrators, owing to the small sample size (*n* = 6) of the group of family homicide perpetrators who did not use mental health services (FH-nMH). Characteristics of the FH-MH and FH-nMH groups are reported in Table 3.

## Discussion

### Main findings

Over two-thirds of perpetrators in our sample (64.5%) had accessed mental healthcare in the period reviewed in the DHR reports. Over half (51.1%) had received a psychiatric diagnosis before the homicide, most commonly depression and anxiety disorders. The high prevalence of depression among IPH perpetrators is consistent with recent systematic reviews of mental disorders and perpetration of intimate partner violence^17^ and general violence.^21^ In our sample, perpetrators of family homicide had higher rates of diagnoses of psychotic disorders; a recent systematic review has shown high rates of past-year patient-reported and relative-reported violence by people with severe mental illness towards relatives, particularly those acting as caregivers, ranging from 19 to 77%.^22^

Perpetrators’ mental health diagnosis or service use do not imply causality between mental disorders and the homicide. A recent study of general population surveys demonstrated an association between a diagnosis of depression and intimate perpetration partner violence, yet the direction of the association was unestablished.^17^ Although serious violence is rare in individuals with a mental health diagnosis, it remains an important adverse outcome for clinical services to consider, as the absolute rates of violent crime over 5–10 years are between 6–10% in individuals with schizophrenia-spectrum disorders.^14^ Comorbid substance misuse or personality disorder increase the risk of IPH in males with a psychiatric diagnosis.^15^

Substance misuse and a history of violence were common overall, particularly among perpetrators who had used mental health services. Alcohol and drug misuse have been associated with intimate partner violence victimisation and perpetration,^23,24^ and three in four men receiving treatment for substance misuse have reported a lifetime history of perpetrating intimate partner violence in a cross-sectional study.^25^

Most perpetrators had a history of DVA perpetration (69.9% of the total sample, and 76.7% in the IPH-MH group); it was less common in the FH-MH group, although still present in more than half of individuals (53.3%). The figure for DVA history includes cases in which it was only revealed to the reporting panel after the homicide had been committed, and in a number of cases such history was not known to healthcare services.

Self-harm or suicide attempts among perpetrators of domestic homicide were common (43.54% in the whole sample, and 60% among perpetrators who had used mental health services), and 21% of the reports involved attempted or completed suicides by the perpetrator after the homicide; the frequency of those incidents was similar across both mental health service-using and non-service-using groups. Studies have reported an increased risk of suicide attempts among perpetrators of family violence in military populations and among male perpetrators of IPH within the criminal justice system.^26,27^ Police data have shown that offenders charged with domestic homicide were three times more likely to have a history of suicidality than individuals arrested for other offences.^28^ However, the direction of causality remain unclear.

There were important differences in the pattern of mental health service use among the IPH and the family homicide groups. Perpetrators of family homicide had much higher rates of psychiatric diagnoses, whereas perpetrators of IPH had higher rates of no diagnosis. Although it is not possible to reach conclusions about the level of risk posed by the different diagnostic categories based on this sample, our findings suggest that perpetrators of family homicide who use mental health services tend to have more enduring mental health problems compared with perpetrators of IPH, and the higher prevalence of use of community mental health teams (66.7% *v.* 30% in the IPH group) and forensic and custody mental health services (16.7% *v.* 5.5% in the IPH group) reflects that characteristic. A higher number of family homicide perpetrators had last used mental health services over 1 year before the homicide (42.8% *v.* 27.2% in the IPH group), although the difference was not statistically significant. Notably, over one in three perpetrators of IPH used mental health services in the month before the homicide.

IPH perpetrators’ higher prevalence of emergency services’ use (31.1% *v.* 16.7% in the family homicide group) and of lack of formal diagnoses or follow-up indicate that their service use tends to be episodic and crisis-driven. This poses challenges when assessing risk, owing to the lack of access to background information and continuity of care. IPH perpetrators were more likely to have a history of DVA perpetration and to have (or be a partner or ex-partner of someone who had) children under the age of 16, which makes it vital that assessments by mental health services include information about partner (and ex-partner) and family, even if mental health follow-up is not required.

### Strengths

To our knowledge, this is the first study to analyse the patterns of mental health service use of perpetrators of domestic homicide using data from DHR reports in England and Wales. By conducting separate analyses of IPH and family homicide, our study added to the current evidence base on these two different types of domestic homicide and the differences between these groups.

### Limitations

We searched for reports systematically in order to minimise the risk of bias in the sampling of reports, but it was not possible to obtain all reports published to date.

DHR reports are written retrospectively with the knowledge that a homicide has been committed. Public inquiries on violence perpetrated by people with mental illnesses have been criticised for hindsight bias.^29^ Additionally, DHR reports are not written for research purposes, and data on ethnicity, age, mental health and substance misuse are often missing.

Our results report on patterns of mental health service use by the subgroup of DVA perpetrators who committed homicide, therefore our findings may not be generalisable to perpetrators of non-fatal forms of DVA.

### Implications for clinical practice and policy

Our findings add to the evidence formulated by NICE guidelines^30^ and statutory guidance for the Domestic Abuse Act 2021,^31^ which strongly support the need for health services to take an assertive role in prevention by identifying patients who are at risk of being victims of DVA. NICE guidelines recommend targeted enquiry about DVA experiences or victimisation for individuals accessing mental health services for anxiety or mood disorders, self-harm, suicidality and substance misuse, but there were no explicit recommendations on enquiry in DVA perpetration.^10^

The evidence from wider research of an association between mental disorders, particularly depression^16^ and substance misuse,^22–24^ and perpetration of intimate partner violence, alongside data from DHR reports and police arrests demonstrating a higher incidence of suicidality among perpetrators of domestic homicide, indicate that perpetrators of DVA may access mental health services during times of crisis. Additionally, there is evidence from qualitative studies that male DVA perpetrators may present to healthcare services seeking help for the consequences of relationship difficulties and may disclose DVA perpetration at times of crisis and when they feel listened to by clinicians.^32–34^ These factors suggest that healthcare services can have an important role in identification of DVA perpetration.

The Linking Abuse and Recovery through Advocacy for Victims and Perpetrators (LARA-VP) resource by King's College London advocates for the assessment of risk of violence and abuse (including emotional abuse and coercive control) towards family members, partners and ex-partners as part of the general violence risk assessment for people presenting to mental health services.^35^ Currently there is little official guidance and few standards for health services in the UK on case finding or other forms of identification of DVA perpetrators, including methods of enquiry and the appropriate response to a disclosure of DVA perpetration. There is an urgent need for further research and development of interventions for perpetrators who use mental health services, which should take into account the variety of diagnoses demonstrated in our sample.

## Data Availability

The authors confirm that the data supporting the findings of this study are available within the article or its supplementary materials.

## About the authors

**Poppy MacInnes** is a junior doctor who studied at GKT School of Medical Education, King's College London, UK. **Marilia A. Calcia** is a Consultant Liaison Psychiatrist at the South London and Maudsley NHS Foundation Trust and a doctoral researcher at the Section of Women's Mental Health, Health Service and Population Research Department, Institute of Psychiatry, Psychology and Neuroscience, King's College London. **Michela Martinuzzi** is a junior doctor who studied at GKT School of Medical Education, King's College London, UK. **Cora Griffin** is a junior doctor who studied at GKT School of Medical Education, King's College London, UK. **Siân Oram** is Head of the Section of Women's Mental Health, Health Service and Population Research Department, Institute of Psychiatry, Psychology and Neuroscience, King's College London. **Louise M. Howard** is Emeritus Professor in Women's Mental Health in the Section of Women's Mental Health, Health Service and Population Research Department, Institute of Psychiatry, Psychology and Neuroscience, King's College London, UK.
